# Psychological Interventions for the Treatment of Patients with Chronic Dermatoses: A Systematic Literature Review

**DOI:** 10.3390/healthcare13222947

**Published:** 2025-11-17

**Authors:** Vera Almeida, Ângela Ferreira, Ana Veloso, Rita Rocha, Ângela Leite, Ana Teixeira

**Affiliations:** 1 UCIBIO—Applied Molecular Biosciences Unit, MedTech—Laboratory of Pharmaceutical Technology, Department of Drug Sciences, Faculty of Pharmacy, University of Porto, Rua de Jorge Viterbo Ferreira 228, 4050-313 Porto, Portugalana.teixeira@iucs.cespu.pt (A.T.); 2 Associate Laboratory i4HB, Institute for Health and Bioeconomy, Faculty of Pharmacy, University of Porto, Rua de Jorge Viterbo Ferreira 228, 4050-313 Porto, Portugal; 3 UNIPRO—Research Unit in Oral Pathology and Rehabilitation, University Institute of Health Sciences (IUCS), CESPU, 4585-116 Gandra, Portugal; 4 University Institute of Health Sciences (IUCS, CESPU, CRL), 4585-116 Gandra, Portugal; 5 Centro de Estudos Filosóficos e Humanísticos, Universidade Católica Portuguesa, Praça da Faculdade 1, 4710-297 Braga, Portugal; 6 Associate Laboratory i4HB, Institute for Health and Bioeconomy, University Institute of Health Sciences, CESPU, 4585-116 Gandra, Portugal; 7 UCIBIO—Applied Molecular Biosciences Unit, Translational Toxicology Research Laboratory, University Institute of Health Sciences (1H-TOXRUN, IUCS-CESPU), 4585-116 Gandra, Portugal

**Keywords:** dermatoses, skin diseases, psychological intervention, self-compassion, body image, atopic dermatitis, psoriasis

## Abstract

**Objectives:** Chronic dermatoses are extremely prevalent and can manifest in various forms across genders and ages. Faced with the symptoms experienced by these conditions and the patient’s perception of the disease and its manifestation, it often leads to isolation and difficulty in emotional regulation. All these symptoms are associated with low quality of life, resulting in depressive and anxious symptomatology. **Methods:** This systematic literature review aims to study psychosocial interventions with an impact on the treatment of patients with chronic dermatoses. Preferred Reporting Items for Systematic Reviews and Meta-Analyses (PRISMA) guidelines were followed, guiding a systematic search across PubMed, Google Scholar, and PsycNet databases. The considered studies reported the impact of interventions when applied to patients with chronic dermatoses. All the studies found were published in peer-reviewed journals. **Results:** The analysis revealed that interventions based on mindfulness, self-compassion, and self-help showed promise, with several studies reporting reductions in disease-related suffering and improvements in quality of life. However, the results were heterogeneous, with some interventions showing no significant benefit over control conditions for specific outcomes like anxiety or appearance-related distress. **Conclusions:** The development of research lines to enhance knowledge in this field will allow for significant improvements in therapeutic care for patients with chronic dermatoses, aiming to support professionals in the development of integrative therapeutic strategies for these patients in their clinical practice.

## 1. Introduction

Society is frequently exposed to live and virtual images that pose a threat to body image, images that are related to appearance and lead to a reduction in body satisfaction or a momentary decrease in evaluative attitude towards one’s own body or its parts. Another aspect can be defined as discomfort or apprehension about body parts falling short of culturally defined beauty standards. A positive body image does not imply the absence of a negative body image; it is a flexible, holistic, and protective construct that goes beyond mere appearance satisfaction to include respect, honor, love, and acceptance of the body, including its unique features that deviate from appearance ideals [1].

The skin, as the most visible organ, is central to body image and self-perception. However, many chronic skin conditions—such as psoriasis, atopic dermatitis, vitiligo, and chronic urticaria—can profoundly disrupt an individual’s relationship with their body [2]. Chronic dermatoses are extremely prevalent and, due to their visible nature, carry a significant psychosocial burden. The self-assessed implications by individuals with a skin condition are more strongly associated with psychological distress than the severity estimated by the physician, suggesting that individuals’ perceptions and emotions regarding their skin condition play a fundamental role in the development of related distress [3].

Broader surveys of conditions affecting appearance report findings where individuals with visible signs tend to experience above-average levels of psychological distress [4,5]. There is considerable individual variation in the psychosocial impact of an altered appearance, and mental health condition can therefore affect the burden, severity, and implications of physical conditions. This highlights the need for attention from healthcare professionals, especially those in the mental health field [3]. In fact, chronic dermatoses are conditions frequently associated with significant psychological impacts, including symptoms of anxiety, depression, and reduced quality of life, as evidenced in the literature emphasizes the importance of addressing these issues through integrated psychological interventions within the clinical management of such conditions, highlighting the need for multidisciplinary approaches that consider both the physical and psychological aspects of the disease [6].

This strong bidirectional link between psychological state and skin health is underpinned by well-characterized biological pathways. Psychological stress activates the hypothalamic–pituitary–adrenal (HPA) axis and the sympathetic nervous system (SNS), leading to the release of neuroendocrine mediators such as cortisol and catecholamines [7]. These substances can directly modulate immune function and promote inflammation, a key driver of many chronic dermatoses like psoriasis and atopic dermatitis [7]. Furthermore, stress can impair the skin’s barrier function and increase pruritus (itching), triggering a scratch-itch cycle that exacerbates lesions [8]. Therefore, psychological interventions that effectively reduce stress may not only alleviate psychological distress but also directly mitigate this neuroimmunocutaneous activity, potentially leading to disease modification.

Consequently, patients often experience symptoms such as shame, distress, frustration, lack of confidence, loss of self-esteem, and body image issues [9], which, combined with the patient’s perception of the illness and its manifestation, often leads to isolation, abandonment of activities, difficulties in relationships with others, and challenges in emotional regulation. In effect, all these symptoms are associated with elevated levels of low quality of life that generate depressive and anxiety-related symptoms [10].

In response to this complex biopsychosocial interplay, psychological interventions for the treatment of dermatoses have been developed, often based on principles of self-compassion, self-concept, and mindfulness, which are rooted in non-self-judgment and acceptance of inner experiences and physical sensations. Mindfulness-based therapies not only aim to increase body awareness and regulate suffering overall but also emphasize the fundamental role of self-compassion in the patient’s ability to be kind to oneself during moments of heightened distress [11]. In addition to these interventions, expressive writing based on self-compassion aims to stimulate self-compassion to improve body image, especially in situations of failure, humiliation, and feelings of loss. This approach seeks to prevent negative thoughts about oneself and one’s body [12]. This intervention has shown promise in other visible conditions; for example, in a study conducted by [13], when applied, expressive writing based on self-compassion in the post-cancer process, there was an observed increase in self-compassion, demonstrating an improvement in distress. The aim of this systematic literature review is to investigate the efficacy of these and other psychological interventions for patients with chronic dermatoses. Based on the existing literature, we expect to find that interventions focusing on mindfulness and self-compassion will be particularly effective in reducing psychological distress and improving quality of life in this patient population.

## 2. Materials and Methods

### 2.1. Search Strategy

This review was conducted by PRISMA (Preferred Reporting Items for Systematic Reviews and Meta-Analyses) guidelines (PRISMA check list are available in the Supplementary Materials—Table S1) and registered on the International Prospective Register of Systematic Reviews (PROSPERO) under the registration number 1170236. The databases selected were PubMed, Google Scholar and PsycNet. The keywords used were: “dermatoses”, “skin diseases”, “psychological intervention”, “self-compassion “, “body image”, “atopic dermatitis” and “psoriasis”. Some studies were obtained through the references of others. All the authors were actively involved in all stages of the review process.

The core search criteria, including the key concepts, search terms, and Boolean operators used, are detailed in Table 1. The search strategy was adapted for the specific syntax of each database.

### 2.2. Inclusion Criteria

The titles and abstracts of the studies were evaluated and selected for inclusion following specific criteria: (a) complete studies (without protocols); (b) studies correlating dermatoses and psychological interventions; (c) involving specific interventions and their psychological and psychosocial outcomes; and (d) studies published in peer-reviewed journals. The articles considered are in English and Portuguese.

### 2.3. Exclusion Criteria

Studies were excluded if they focused only on dermatoses and excluded psychological interventions, or if they addressed only the dermatological or pharmacological aspects themselves and did not establish an association with the psychological dimensions. Studies that addressed interventions with the family were also excluded, as were case reports and studies that did not describe the results of the intervention and had a small sample size (<10 participants).

### 2.4. Screening

The title/summary of the studies was selected independently, always based on the inclusion and exclusion criteria.

### 2.5. Quality Assessment

The methodological quality and risk of bias of the included studies were critically appraised using the appropriate Joanna Briggs Institute (JBI) checklists for study design (2024) [14]. The appraisal was conducted independently by the review authors, with any discrepancies resolved through consensus. The results of this assessment are summarized in Table 2, Table 3, Table 4, Table 5 and Table 6. The overall risk of bias for each study, as determined by this appraisal, was considered when interpreting the results and drawing conclusions in this review.

**Table 2:** Randomized Controlled Trials.

Authors	Q1	Q2	Q3	Q4	Q5	Q6	Q7	Q8	Q9	Q10	Q11	Q12	Q13	% Yes	Risk of Bias
(Adkins, 2021) [15]	Y	U	Y	U	NA	Y	Y	N	Y	Y	Y	Y	Y	69.2	Moderate
(Kelly et al., 2009) [16]	Y	U	Y	N	NA	NA	Y	Y	Y	Y	Y	Y	Y	69.2	Moderate
(D’Alton et al., 2019b) [17]	Y	U	N	N	U	U	Y	Y	U	Y	Y	Y	Y	53.8	Moderate
(Singh et al., 2017) [18]	Y	U	U	N	U	U	Y	Y	Y	Y	Y	Y	Y	61.5	Moderate
(Larsen et al., 2014b) [19]	Y	U	U	N	U	U	Y	Y	U	Y	Y	Y	Y	53.8	Moderate
(Kishimoto et al., 2023) [20]	Y	Y	Y	N	N	Y	Y	Y	Y	Y	Y	Y	Y	84.6	Low
(Hudson et al., 2020) [21]	Y	U	U	N	N	U	Y	N	Y	Y	Y	Y	Y	53.8	Moderate
(Mifsud et al., 2021b) [22]	Y	Y	Y	Y	NA	Y	Y	Y	Y	Y	Y	Y	Y	92.3	Low
(Muftin et al., 2022) [23]	Y	Y	Y	N	Y	Y	Y	Y	Y	Y	Y	Y	Y	92.3	Low
(Łakuta, 2022) [24]	Y	Y	Y	Y	Y	Y	Y	Y	Y	Y	Y	Y	Y	100	Low
(Seekis et al., 2017b) [1]	Y	U	Y	N	Y	Y	Y	Y	Y	Y	Y	Y	Y	84.6	Low
(Sengupta et al., 2025) [25]	Y	U	U	N	N	U	Y	U	Y	Y	Y	Y	Y	53.8	Moderate
(Sherman et al., 2019) [26]	Y	U	Y	Y	NA	NA	Y	Y	Y	Y	Y	Y	Y	76.9	Low
(Borimnejad et al., 2015) [27]	Y	U	Y	N	N	Y	Y	Y	Y	Y	Y	Y	Y	76.9	Low
(Bundy et al., 2013) [28]	Y	U	Y	U	NA	NA	Y	Y	Y	Y	Y	Y	Y	69.2	Moderate
(Pascual-Sánchez et al., 2020) [29]	Y	U	Y	N	NA	U	Y	NA	Y	Y	Y	Y	Y	61.5	Moderate

Note. 1. Was true randomization used for assignment of participants to treatment groups? 2. Was allocation to treatment groups concealed? 3. Were treatment groups similar at the baseline? 4. Were participants blind to treatment assignment? 5. Were those delivering treatment blind to treatment assignment? 6. Were outcomes assessors blind to treatment assignment? 7. Were treatment groups treated identically other than the intervention of interest? 8. Was follow-up complete and if not, were differences between groups in terms of their follow-up adequately described and analyzed? 9. Were participants analyzed in the groups to which they were randomized? 10. Were outcomes measured in the same way for treatment groups? 11. Were outcomes measured in a reliable way? 12. Was appropriate statistical analysis used? 13. Was the trial design appropriate, and any deviations from the standard RCT design (individual randomization, parallel groups) accounted for in the conduct and analysis of the trial?; U = Unclear; Y = Yes; N = No; NA = Not applicable.

**Table 3:** Quasi-Experimental Studies.

Authors	Q1	Q2	Q3	Q4	Q5	Q6	Q7	Q8	Q9	% Yes	Risk of Bias
(Hedman-Lagerlöf et al., 2019) [30]	Y	N	NA	NA	Y	Y	U	Y	Y	55	Moderate
(Offenbächer et al., 2021) [31]	Y	N	NA	NA	Y	Y	Y	Y	Y	66.6	Moderate
(Harfensteller, 2022) [32]	Y	N	NA	NA	Y	Y	Y	Y	Y	66.6	Moderate
(Ridge et al., 2021) [33]	Y	N	Y	Y	Y	Y	Y	NA	Y	77.7	Low
(Latifi et al., 2020) [34]	Y	Y	Y	Y	Y	Y	Y	Y	Y	100	Low
(Li et al., 2020) [35]	Y	Y	Y	Y	Y	Y	Y	U	Y	88.8	Low

Note. 1. Is it clear in the study what the “cause” and the “effect” are (i.e., there is no confusion about which variable comes first)? 2. Was there a control group? 3. Were participants included in any comparisons similar? 4. Were the participants included in any comparisons receiving similar treatment/care, other than the exposure or intervention of interest? 5. Were there multiple measurements of the outcome, both pre and post the intervention/exposure? 6. Were the outcomes of participants included in any comparisons measured in the same way? 7. Were outcomes measured in a reliable way? 8. Was follow-up complete and if not, were differences between groups in terms of their follow-up adequately described and analyzed? 9. Was appropriate statistical analysis used?; U = Unclear; Y = Yes; N = No; NA = Not applicable.

**Table 4:** Qualitative Research.

Authors	Q1	Q2	Q3	Q4	Q5	Q6	Q7	Q8	Q9	Q10	% Yes	Risk of Bias
(Da Silva et al., 2011) [36]	Y	Y	Y	Y	Y	Y	Y	Y	Y	Y	100	Low
(Zucchelli et al., 2021) [37]	Y	Y	Y	Y	Y	Y	U	Y	Y	Y	90	Low

Note. 1. Is there congruity between the stated philosophical perspective and the research methodology? 2. Is there congruity between the research methodology and the research question or objectives? 3. Is there congruity between the research methodology and the methods used to collect data? 4. Is there congruity between the research methodology and the representation and analysis of data? 5. Is there congruity between the research methodology and the interpretation of results? 6. Is there a statement locating the researcher culturally or theoretically? 7. Is the influence of the researcher on the research, and vice versa, addressed? 8. Are participants, and their voices, adequately represented? 9. Is the research ethical according to current criteria or, for recent studies, and is there evidence of ethical approval by an appropriate body? 10. Do the conclusions drawn in the research report flow from the analysis, or interpretation, of the data?; U = Unclear; Y = Yes.

**Table 5:** Systematic Review.

Authors	Q1	Q2	Q3	Q4	Q5	Q6	Q7	Q8	Q9	Q10	Q11	% Yes	Risk of Bias
(Bartholomew et al., 2022) [38]	Y	Y	Y	U	U	Y	Y	Y	N	Y	Y	72.7	Moderate
(Rafidi et al., 2022) [39]	Y	Y	Y	Y	Y	U	U	Y	N	Y	Y	72.7	Moderate

Note. 1. Is the review question clearly and explicitly stated? 2. Were the inclusion criteria appropriate for the review question? 3. Was the search strategy appropriate? 4. Were the sources and resources used to search for studies adequate? 5. Were the criteria for appraising studies appropriate? 6. Was critical appraisal conducted by two or more reviewers independently? 7. Were there methods to minimize errors in data extraction? 8. Were the methods used to combine studies appropriate? 9. Was the likelihood of publication bias assessed? 10. Were recommendations for policy and/or practice supported by the reported data? 11. Were the specific directives for new research appropriate?; U = Unclear; Y = Yes; N = No.

**Table 6:** Textual Evidence: Expert Opinion.

Authors	Q1	Q2	Q3	Q4	Q5	Q6	% Yes	Risk of Bias
(Yosipovitch et al., 2024) [8]	Y	Y	Y	U	Y	Y	83.3	Low

Note. 1. Is the source of the opinion clearly identified? 2. Does the source of the opinion have standing in the field of expertise? 3. Are the interests of the relevant population the central focus of the opinion? 4. Does the opinion demonstrate a logically defended argument to support the conclusions drawn? 5. Is there reference to the extant literature? 6. Is any incongruence with the literature/sources logically defended?; U = Unclear; Y = Yes.

## 3. Results

**Figure 1:**
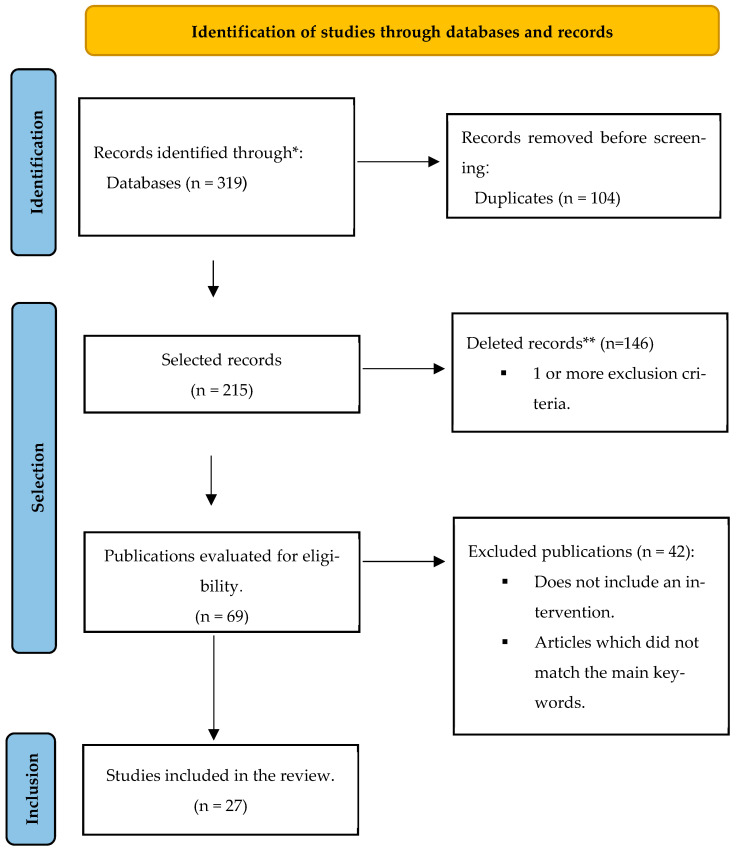
PRISMA flow diagram comprising the different phases of this systematic review. * Refers to records identified through database searching (PubMed, Google Scholar, PsycNet). ** Records deleted for meeting one or more predefined exclusion criteria.

The initial searches in the databases yielded a total of 319 articles, of which 104 (32.60%) were removed due to duplication. The abstracts of the remaining 215 (67.39%) studies were analyzed, and 146 (45.77%) were excluded. Among the remaining 69 (21.63%) full-text articles, 42 (13.17%) were excluded, resulting in 27 (8.46%) articles for review (Figure 1).

### 3.1. Studies’ Characteristics

The characteristics of the studies (n = 27) are presented in Table 7. Three studies (11.11%) included cancer survivors, twenty-three studies (85.19%) focused on patients with dermatological conditions, predominantly psoriasis, and one study (3.70%) addressed the role of negative body image in individuals with dermatoses. Of the 27 studies, four (14.81%) applied the intervention of expressive writing based on self-compassion, and nineteen (70.37%) applied interventions based on mindfulness therapy. Regarding methodology, twelve (44.44%) were randomized controlled trials, five (18.52%) randomized study, two (7.41%) were pilot studies, five (7.41%) were qualitative studies, one (3.70%) was a prospective study; one (3.70%) was a prospective cutting study, and one (3.70%) was an open trial.

### 3.2. Studies’ Results Summary

In the analysis of the tables (Table 8 and Table 9), we observe that the selected studies present a similar methodology, twelve (44.44%) were randomized controlled trials, five (18.52%) randomized study, two (7.41%) were pilot studies, five (7.41%) were qualitative studies, one (3.70%) was a prospective study; one (3.70%) was a prospective cutting study, and one (3.70%) was an open trial. As seen in Table 9, despite the variations in the instruments used to assess the effectiveness of interventions across different studies, they generally evaluated depression and anxiety [12,16,17,18,20,22,25,28,29,31,33,35], quality of life [17,18,20,23,24,25,28,29,31,35,38], self-compassion [12,20,22,26,27], and body appreciation [1,12,22,24,26,34]. The most studied variables included sociodemographic characteristics, with individuals diagnosed with chronic dermatoses, predominantly psoriasis and vitiligo (Table 8). The articles are grounded in aspects related to self-compassion, self-help, body image, and quality of life in patients with dermatoses. Most of the studies are classified as Q1 and Q2 metrics, indicating the significance and recognition of research on the subject.

The evidence for expressive writing was mixed. While a general writing intervention on body functionality showed no significant effects on appearance anxiety or skin-related quality of life [15,26], writing tasks specifically designed to cultivate self-compassion demonstrated more consistent benefits for body appreciation and satisfaction [1,15] compared to control conditions. These data can be explained by the fact that expressive writing based on self-compassion requires more awareness, greater self-reflection, and a stronger self-connection [32], as kindness and unconditional acceptance are also important for body acceptance. These results can be useful for clinical use since cultivating a self-compassionate attitude toward stressors can promote better coping and improve mood [32].

Mind–Body therapies (MBT) in atopic dermatitis (AD) demonstrated promising effects on both physical and psychological outcomes. Studies reported reductions in pruritus, scratching behaviors, and overall disease severity, alongside improvements in anxiety, stress, depressive symptoms, and quality of life. Interventions including mindfulness, cognitive–behavioral therapy, hypnotherapy, relaxation techniques, biofeedback, and therapeutic massage showed beneficial effects as adjuncts to conventional treatment [8]. Mindfulness and self-compassion–based interventions significantly reduced psychological distress in individuals with chronic skin conditions. Improvements were observed in depression, anxiety, stress, self-esteem, dermatology-related quality of life, and overall well-being. Mindfulness enhanced emotional regulation and reduced rumination, self-judgment, and experiential avoidance. Self-compassion promoted acceptance, self-kindness, and recognition of the universality of negative experiences. These findings suggest that such interventions may be effective as adjuncts in psychodermatological care [25,40].

In mindfulness-based interventions [3,17,25,31,32,33,38], a decrease in the severity of symptomatology, as well as emotional suffering caused by the physical effects of dermatoses, was observed. Improvements in stress, changes in depressive symptomatology (but not anxiety), and enhanced coping with the disease were also noted. The studies reported that mindfulness led to improvements in symptoms, coping mechanisms, a deeper understanding of emotions, increased awareness of impulses, and enhancements in positive psychological attributes. In another study comparing mindfulness-based interventions such as Mindfulness-Based Cognitive Therapy, Mindfulness-Based Self-Compassion Therapy, and self-help to usual treatment, despite the beneficial aspects of these interventions, they were not significant for psychological well-being. The evidence on meditation and mindfulness practices in psoriasis is promising, but still limited by the small number of RCTs and short follow-up periods. Most studies reported improvements in psoriasis severity measured by the saPASI, while only a few demonstrated significant effects on quality of life. These interventions may also address psychological comorbidities, such as anxiety, depression, and worry, providing additional benefits beyond physical symptom relief [38].

In two studies, Cognitive–Behavioral Therapy (CBT) was used; in one, exposure-based CBT involving mindfulness practice was employed [30], and in another, general CBT was applied to dermatosis [28]. In both studies, a reduction in anxiety and improvements in quality of life were observed, but there were no changes in the depressive component. In mindfulness-based CBT, quality of life underwent changes during the intervention, while in the study of general CBT applied to dermatosis, the results regarding quality of life were more significant. Perhaps this difference is due to the latter intervention having a greater focus on overall disease management rather than specifically targeting the treatment of suffering for psoriasis. In both studies, careful consideration is warranted when analyzing results due to the potentially limiting small sample size for interventions based on self-compassion and Mindfulness [20,22,26,27,34], various studies reported significant effects on stress, self-compassion, anxiety, and depression [21]. These interventions assisted in the acceptance of altered appearance [37], reduction in shame and skin complaints, and a greater reduction in depression was observed in individuals with higher levels of self-criticism [16]. In one study [31], with a similar intervention basis, an increase in anxiety and depression levels was reported; however, caution is advised due to the small sample size, which prevents definitive conclusions.

Finally, in a study where individual motivational interviewing was used [19], significant changes were observed in lifestyle and overall positive changes. However, this intervention is recommended as a potential complement to medical management and for patient education regarding the condition. In a self-affirmation intervention [24], despite significant results for depressive symptoms, anxiety, and well-being, no differences were observed in mental health. The conclusion reached was that self-affirmation is not a pathway to improving psychological functioning in patients with dermatoses.

**Table 8:** Variables, analysis, and statistical methods used in the studies reviewed for this literature.

Reference	Analysis and Statistical Methods	Variables
(Adkins, 2021) [15]	ANCOVA; Cronbach’s alphas	Age; gender; ethnicity, educational; dermatological condition that affects their body image; language
(Ahmed et al., 2018) [9]	Independent *t*-tests	People with vitiligo; age (>18)
(Bartholomew et al., 2022) [38]	Qualitative synthesis	Type of intervention; psoriasis; Psoriasis Area and Severity Index; Dermatology Life Quality Index; Perceived stress
(Borimnejad et al., 2015) [27]	Student’s *t*-tests;	Age; diagnosis of vitiligo confirmed; ability to read and write
(Bundy et al., 2013) [28]	Analise of covariance (ANCOVA), intention-to treat (ITT), multiple imputation, multivariate logistic regression, Shapiro–Wilk test, Stata v12	
(Clarke et al., 2020b) [3]	SPSS 26; multiple regression; bivariate correlations; independent *t*-tests	Dermatology patients; age; gender; ethnicity; employment status; marital status; education level
(D’Alton et al., 2019b) [17]	Not discriminated	Age; diagnosis of psoriasis; systemic medication for 6 months or more
(Da Silva et al., 2011) [36]	Not discriminated	People with psychodermatoses; age; gender
(Galhardo et al., 2022) [7]	SPSS, v. 27; Pearson’s correlation; hierarchical multiple linear regression; Durbin–Watson statistics;	People with a diagnosis of psoriasis; age; gender
(Harfensteller, 2022) [32]	Spearman’s correlation coefficient; SPSS IBM 26; *t*-tests; Cohen’s d;	Patients with diagnosed Atopic dermatitis (AD); age (18–65); language
(Hedman-Lagerlöf et al., 2019) [30]	STATA version 14.2; *t*-tests;	Age (18–65); adults with Atopic dermatitis; duration of AD for at least 6 months; language
(Hewitt et al., 2022) [41]	NVivo 12 Pro;	Age; self-diagnosed dermatological condition
(Hudson et al., 2020) [21]	Independent samples *t*-tests; chi square tests	Age (16); English-speaking; diagnosis of a skin condition;
(Hughes et al., 2023) [42]	Thematic analysis	8–11 years of age; diagnosed with any skin condition and English-language speakers; eligible parents were 18 years of age or over; the child’s main caregiver
(Kelly et al., 2009) [16]	ANOVAs;	Age; facial acne; prescribed acne treatment perceived to be ineffective
(Kishimoto et al., 2023) [20]	Mixed model for repeated measures (MMRM), adjusting for age, sex, and baseline DLQI, to assess within- and between-group differences. Additional DLQI analyses included: (1) percentage of patients with >4-point improvement, (2) subgroup analysis by sex, age, and baseline DLQI, and (3) per-protocol comparisons. All analyses used SAS 9.4 with standard corrections	Age, sex, education, marital status, living situation, working situation Disease duration, dermatologic treatment
(Łakuta, 2022) [24]	Six linear mixed models (LMMs); PROCESS macro version 3.5.3;	Age; physician-diagnosed psoriasis
(Larsen et al., 2014b) [19]	SPSS version 19; *t*-tests; qui 2 statistics, or Mann–Whitney U tests; ANOVA; Cohen’s d; ANCOVAs;	Age; gender; educational level; health status; disease duration
(Latifi et al., 2020) [34]	Descriptive statistics (frequency, percentage, mean, and standard deviation) and inferential statistics (repeated-measures ANOVA and Kruskal–Wallis test)	Women with skin cancer; age; children; education. Checklist of physiological symptoms related to skin cancer: skin changes, unreasonable weight loss, bloating, change in the chest, abnormal bleeding, trouble with swallowing, blood in the stool, abdominal pain, depression, mouth infections, persistent and unspecified pain, changes in lymph nodes, fever, fatigue, persistent cough, and indigestion. Three scales (severe, partial, and never) to assess the symptoms, and patients reported changes and a lack of changes in their status.
(Li et al., 2020) [35]	SPSS Statistics for Windows, Version 17.0. Student’s *t*-tests and the chi-square tests; hospitalization length and patient satisfaction between the different groups.	Age, gender, mean time from diagnosis to treatment initiation, and family history of psoriasis
(Melissant et al., 2021b) [12]	SPSS version 26; Multiple regression model; Linear mixed;	Head and neck cancer (HNC) survivors
(Mifsud et al., 2021b) [22]	c2 tests of Independence; ANOVA; chi-square tests; Shapiro–Wilk’s; Levene’s Test of Homogeneity of Variance; SPSS version 23;	Age; gender; diagnosed with stage I to III breast cancer, ductal carcinoma in situ (DCIS) and/or lobular carcinoma in situ (LCIS); experienced at least one negative event related to the changes that have occurred to their body after breast cancer; language
(Muftin et al., 2022) [23]	SPSS Statistics; intention-to-treat (ITT); v2-tests; MANOVA; ANOVA;	Gender; age; ethnicity; education
(Offenbächer et al., 2021) [31]	SPSS; *t*-test; per-protocol analysis (PPA);	Age; diagnosis of AD;
(Pascual-Sánchez et al., 2020) [29]	IBM SPSS Statistics for Macintosh, Version 21.0; *t*-test for independent sample; Pearson correlation	Women with AAU; age; time of disease; number of received treatments
(Rafidi et al., 2022) [39]	Qualitative synthesis	Type of intervention; dermatologic disease; treatment outcomes
(Ridge et al., 2021) [33]	GraphPad Prism software; version 9.3.1.	Age; diagnosis of chronic urticaria
(Seekis et al., 2017b) [1]	MANOVA; one-way ANOVA;	Age (17–25); language
(Sengupta et al., 2025) [25]	SPSS version 27; ANCOVAs	Age; Depression; Anxiety; Stress; Dermatology-specific quality of life; Self-esteem; Well-being
(Sherman et al., 2019) [26]	SPSS version 25.0; Chi-square; *t*-test; R statistics software version 4.5.2; ANCOVAs	Age, gender, education level, skin condition type, time since skin condition onset; whether treatment was received for the skin condition
(Singh et al., 2017) [18]	SPSS version 18; Wilcoxon signed-rank test	Age (>15); moderate and severe chronic plaque psoriasis
(Yosipovitch et al., 2024) [8]	Focused literature review of mind–body therapies	Pruritus/itch; pain; stress; sleep disturbances; anxiety; depressive symptoms; dermatology-specific quality of life; scratching behavior
(Zucchelli et al., 2021) [37]	NVivo© version 15.3.0 software;	Age; gender; participants with a range of appearance-affecting conditions; language

**Table 9:** Instruments used in the studies reviewed for this review.

Reference	Instruments
(Borimnejad et al., 2015) [27]	General Health Questionnaire-28 (GHQ-28);
(Bundy et al., 2013) [28]	Hospital Anxiety Depression Scale (HADS) Self-Administered Psoriasis Area and Severity Index (SAPASI) Dermatology Life Quality Index (DLQI) Illness Perception Questionnaire
(D’Alton et al., 2019b) [17]	The Hospital Anxiety and Depression Scale (HADS); The Penn State Worry Questionnaire (PSWQ); The Five Facet Mindfulness Questionnaire (FFMQ); The Fears of Compassion Scales (FCS); The World Health Organization Quality of Life-BREF (WHOQOLBREF); The Dermatology Life Quality Index (DLQI); The Psoriasis Area and Severity Index (PASI);
(Kelly et al., 2009) [16]	Depressive Experiences Questionnaire (DEQ); The Beck Depression Inventory (BDI); Experiences of Shame Scale (ESS); SKINDEX-16;
(Kishimoto et al., 2023) [20]	Dermatology Life Quality Index—Japanese version (DLQI-J) Patient-Oriented Eczema Measure (POEM) Japanese version Evaluation of itching Self-Compassion Scale—Japanese version SCS-J Japanese version of the Freiburg Mindfulness Inventory (FMI) Hospital anxiety and depression scale (HADS)—Japanese version Japanese version of the Internalized Shame Scale (Japanese version of the ISS) Dermatological treatment adherence Home practice record
(Łakuta, 2022) [24]	Health Questionnaire [PHQ-9]; Mental Health Continuum—Short Form [MHCSF]; Self-Affirming Implementation Intention (S-AII); Body-Related Self-Affirming Implementation Intention (BS-AII);
(Larsen et al., 2014b) [19]	Self-Administered Psoriasis Area and Severity Index (SAPASI); Self-management measured (heiQ); The Psoriasis Knowledge Questionnaire (PKQ); The Brief Illness Perception Questionnaire (BIPQ);
(Latifi et al., 2020) [34]	Self-compassion scale (SCS) Body Image Concern Inventory (BICI)
(Li et al., 2020) [35]	Symptom Checklist-90 (SCL-90) Self-Rating Depression Scale (SDS) Self-Rating Anxiety Scale (SAS) The Generic Quality of Life Inventory (GQOLI)
(Melissant et al., 2021b) [12]	Body Image Scale (BIS); Body Appreciation Scale (BAS-2); Self-Compassion Scale—Short Form (SCS-SF); Hospital Anxiety and Depression Scale (HADS), (HADS-A), (HADS-D); Female Sexual Function Index (FSFI-6); International Index of Erectile Function (IIEF-5);
(Mifsud et al., 2021b) [22]	Body Image Scale (BIS: [43]); The Body Appreciation Scale; Self-compassionate attitude (SCA); Self-Compassion Scale—Short Form (SCS-SF); Positive and Negative Affect Schedule (PANAS); Short form of the Depression, Anxiety and Stress Scale (DASS21);
(Muftin et al., 2022) [23]	Other as Shamer Scale (OAS); The Forms of Self-Criticizing/Attacking & Self-Reassuring Scale (FSCRS); The Dermatology Life Quality Index (DLQI);
(Offenbächer et al., 2021) [31]	Score of Atopic Dermatitis (SCORAD); Patient Oriented Eczema Measure (POEM); Dermatology Life Quality Index (DLQI); Hospital Anxiety and Depression Scale (HADS); Perceived Stress Questionnaire (PSQ); Freiburger Mindfulness Inventory (FMI); Mindful Attention Awareness Scale (MAAS); Global Transition Items;
(Pascual-Sánchez et al., 2020) [29]	Dermatology Life Quality Index—DLQI Beck Depression Inventory—BDI State-trait Anxiety Inventory—STAI Rosenberg Self-esteem Scale—RSES Toronto Alexithymia Scale—TAS-20
(Ridge et al., 2021) [33]	Depression and Anxiety Stress Scale (DASS 21); the Five Facets of Mindfulness Questionnaire (FFMQ); Urticaria Control Test; PERMA profiler;
(Seekis et al., 2017b) [1]	State Body Appreciation Scale-2 (SBAS-2); Body Image States Scale (BISS);
(Sengupta et al., 2025) [25]	Dermatology Life Quality Index (DLQI) Rosenberg Self-Esteem Scale (RSES) World Health Organization Well-Being Index (WHO-5) Depression, Anxiety, and Stress Scale (DASS-21)
(Sherman et al., 2019) [26]	Self-Compassion Scale—Short Form (SCS-SF); Positive and Negative Affect Schedule (PANAS); Body Image Disturbance Questionnaire;
(Singh et al., 2017) [18]	Psoriasis Area Severity Index (PASI); Dermatology Life Quality Index (DLQI); WHO-5 well-being index (WHO-5); Patient health questionnaire (PHQ); Generalized anxiety disorder (GAD)-7;
(Zucchelli et al., 2021) [37]	Not discriminated

## 4. Discussion

The objective of this literature review is to investigate psychological interventions for the treatment of chronic dermatoses. Chronic dermatoses, especially psoriasis and vitiligo, have a significant impact on an individual’s life, leading to depression, anxiety, an increased risk of suicide, experiences of stigma, tendencies towards isolation, and negative body image [44]. All these factors can affect the individual’s quality of life [45] and may pose a risk of psychiatric morbidity [9]. The difficulties faced by individuals with dermatoses regarding their self-image and social life are mentioned [46]. In this sense, psychological intervention is essential.

Among the interventions used in different articles, expressive writing-based intervention has the potential to promote a positive body image and increase self-compassion. Interventions based on Mindfulness, self-compassion, and self-help demonstrate greater efficacy in the treatment of chronic dermatoses and can be a complementary tool for intervention [47]. They are promising in addressing the psychological stress generated by the effects of chronic dermatoses on the skin, mitigating negative thoughts about body image, reducing feelings of shame, skin complaints, depression, and self-criticism, and improving quality of life levels [48]. These interventions were also reported with higher participant satisfaction. While interventions based on Cognitive–Behavioral Therapy (CBT) show positive results, their practice is general and non-specific, demonstrating significant effects only when applied in conjunction with mindfulness practices.

The success of mindfulness-based and self-compassion interventions in reducing disease-related suffering and improving quality of life can be understood within the context of the neuroimmunocutaneous framework discussed in the introduction. By fostering a non-judgmental awareness of thoughts and sensations (mindfulness) and cultivating self-kindness during distress (self-compassion), these techniques can downregulate the maladaptive stress response [49,50]. This psychophysiological shift likely leads to a reduction in pro-inflammatory signaling and a break in the stress-itch cycle [8,39]. Consequently, the observed benefits of these interventions may extend beyond the psychological realm, contributing to a direct, positive impact on disease activity—such as reducing flare-ups, severity of lesions, and pruritus—by modulating the very biological pathways that link the mind and the skin. This underscores the potential of psychosocial strategies as integral components of a treatment plan aimed at comprehensive disease management.

A critical appraisal of the evidence base reveals several recurrent limitations within the included studies, which consequently inform the constraints of the present review. The primary literature is frequently characterized by modest sample sizes and notable attrition rates, which challenge the statistical power and generalizability of the findings. Furthermore, the context of participant recruitment introduces potential bias; for instance, individuals recruited in clinical settings may present with lower symptom severity and demonstrate reduced post-treatment improvement, suggesting that interventions might yield different effects for those experiencing higher levels of distress. The interpretability of results is also complicated by confounding variables, such as the concurrent use of antidepressant medication and the fluctuating nature of dermatoses, where the level of disease exacerbation at the time of assessment can significantly influence outcomes. An additional methodological concern across studies is the predominant reliance on self-report measures for psychological outcomes, which inherently carries the potential for bias. These inherent limitations in the primary literature directly shape the constraints of this review. While conducted according to PRISMA guidelines, the present synthesis was limited by its search strategy, being restricted to three databases and including only English and Portuguese publications, which may have led to the omission of pertinent research. The significant heterogeneity observed in the interventions, methodological designs (ranging from RCTs to qualitative studies), and outcome measures precluded a quantitative meta-analysis, thereby limiting the ability to draw definitive conclusions regarding efficacy. Finally, the broad focus on chronic dermatoses as a whole means the findings may not be uniformly applicable to each specific condition. Collectively, these factors underscore that the conclusions drawn here, while promising, should be viewed as indicative of a rapidly evolving field rather than as definitive evidence.

Considering the objective of this study, the analysis of the articles easily reveals the understanding and knowledge that experts have regarding the physical, psychological, and social impact of chronic dermatoses on an individual’s life. However, the therapists’ knowledge for assisting these patients is limited when it comes to determining the most suitable intervention. Despite the promising results observed in the studies, it would be important to develop more objective research with conclusive outcomes to enable better adaptation to the characteristics of the patient, as well as the use of feasible interventions.

The purpose of this literature review was to clarify which interventions demonstrate effectiveness in the treatment of patients with chronic dermatoses, with the aim of increasing knowledge in this field.

In future research, it would be important to conduct comparative studies between different psychological interventions to determine relative effectiveness, patient acceptability, and long-term outcomes. Evaluating the effectiveness of different intervention formats, such as in-person sessions, online videos, and remote interventions, is crucial to understanding the feasibility and efficacy of these approaches in different contexts and populations. Given the limited sample sizes and high dropout rates, it would be important to investigate factors influencing patient acceptance and participation in psychological interventions, considering barriers such as stigma, accessibility, and individual preferences. Finally, delving deeper into the relationship between improvements in dermatoses severity measures and the reduction in psychological distress would be a valuable avenue for exploration. Furthermore, the emergence of telehealth and digital health platforms presents a transformative prospect for expanding the reach and accessibility of psychological interventions. Future research should rigorously evaluate the efficacy and long-term adherence of interventions delivered remotely via videoconferencing, mobile applications, and structured online programs. Investigating how these digital formats compare to traditional in-person therapy in terms of clinical outcomes, patient engagement, and economic feasibility will be crucial. Embracing telemedicine could ultimately help overcome barriers such as geographic limitations, mobility issues, and stigma, making integrative psychodermatological care available to a broader and more diverse patient population.

## 5. Conclusions

The psychological impact of chronic dermatoses is well-documented in the literature, significantly affecting patients’ self-esteem, body image, and overall quality of life. In this context, psychological interventions that address not only the physical symptoms but also the emotional and social consequences of skin disorders are essential.

The evidence reviewed in this study suggests that therapeutic approaches based on mindfulness, self-compassion, and self-help are promising but heterogeneous. While they show potential for managing psychological distress and improving quality of life in patients with chronic dermatoses, their effectiveness varies across specific outcomes, conditions, and intervention formats. Future research should aim to identify the most effective components of these interventions and the patient populations most likely to benefit. Additionally, expressive writing interventions focused on self-compassion have shown potential in promoting body image acceptance and reducing self-critical thoughts.

In summary, this review contributes to the growing body of knowledge on psychological interventions for chronic dermatoses and underscores the importance of an interdisciplinary approach that integrates both physical and psychological aspects in the care of dermatological patients.

## Figures and Tables

**Table 1:** Research strategy.

Database	Research Strategy
PubMed	(“Skin Diseases” [Mesh] OR dermatos * OR “atopic dermatitis” OR eczema OR psoriasis OR vitiligo) AND (“Psychotherapy” [Mesh] OR “Psychosocial Intervention” OR psychotherapy * OR “cognitive behavioral therapy” OR mindfulness OR “acceptance and commitment therapy” OR self-compassion OR “body image” OR “support group *”)
Google Scholar	(“atopic dermatitis” OR eczema) (psychotherapy OR mindfulness OR “cognitive behavioral therapy”) (psoriasis OR vitiligo) (“psychological intervention” OR “support group” OR “body image”) (“skin disease” OR dermatosis) (psychotherapy OR “acceptance and commitment therapy” OR self-compassion)
PsycNet	(DE “Dermatological Disorders” OR TI (dermatos * OR “skin disease *” OR “atopic dermatitis” OR eczema OR psoriasis OR vitiligo) OR AB (dermatos * OR “skin disease *” OR “atopic dermatitis” OR eczema OR psoriasis OR vitiligo)) AND (MH “Psychotherapy+” OR MH “Cognitive Behavior Therapy” OR MH “Mindfulness” OR MH “Body Image” OR DE “Psychosocial Interventions” OR DE “Support Groups” OR TI (psychotherapy * OR “psychological intervention” OR “cognitive behavioral therapy” OR mindfulness OR “body image”) OR AB (psychotherapy * OR “psychological intervention” OR “cognitive behavioral therapy” OR mindfulness OR “body image”))

**Table 7:** Resume of the studies reviewed for this literature review.

Study Title	Reference and Metrics 1	Main Aim	Methodology	Psychological Intervention	Main Results	Discussion/Conclusions	Key Outcomes & Effects
**Self-help targeting body image among adults living with dermatological conditions: An evaluation of a brief writing intervention**	(Adkins, 2021) [15] Q1 (high)	Dermatological conditions can affect how individuals feel about their bodies. This research therefore seeks to evaluate the potential for a brief writing intervention, focused on body functionality, to improve body image in adults living with a range of dermatological conditions.	Randomized Controlled Trial	One week writing intervention, focused on body functionality, to improve body image	For participants with relatively low or mid-range scores on baseline body appreciation and functionality appreciation, there were medium-to-large effects of the intervention. Effects were smaller, with all but-one remaining significant at one-month follow-up and in intention-to-treat analyses. No effects of the intervention were found for measures of appearance anxiety, skin-related shame, and skin-related quality-of-life.	One-week writing intervention has the potential to improve positive aspects of body image, but not appearance and skin-related distress in adults living with a dermatological condition. However, these findings should be considered in the context of high attrition across both the intervention and control conditions.	Medium-to-large improvements in body and functionality appreciation (*p* < 0.001); no effects on appearance anxiety, skin shame, or dermatology-related quality of life.
**Mindfulness and Meditation for Psoriasis: A Systematic Review**	(Bartholomew et al., 2022) [38] Q1 (high)	The aim of this review is to provide an update on research studies investigating the role of mindfulness and meditation in treating psoriasis symptoms, severity, and quality of life.	Systematic review	Guided meditation and mindfulness-based interventions were used to reduce psoriasis severity and improve quality of life	Among six randomized controlled trials (RCTs) included, five showed improvement in psoriasis severity measured by the self-administered Psoriasis Area and Severity Index (saPASI) after 8 to 12 weeks of guided meditation. Additionally, one RCT and one non-randomized controlled trial reported mental health benefits, including reduced anxiety and depression, following mindfulness and meditation interventions	The findings suggest that mindfulness and meditation are promising non-pharmacological interventions for improving both psoriasis severity and patients’ quality of life in the short term	Five of six RCTs reported significant between-group improvements in psoriasis severity (saPASI) following 8–12 weeks of mindfulness or meditation; psychological outcomes (stress, quality of life, anxiety, depression, mindfulness) showed limited or inconsistent between-group effects; long-term effects remain unclear.
**The Effect of Expressive Writing on Psychological Distress in Patients with Vitiligo: A Randomized Clinical Trial**	(Borimnejad et al., 2015) [27] Q2 (medium)	Assess whether expressive writing, as a psychological intervention, reduces psychological distress in vitiligo patients undergoing phototherapy.	Randomized study.	Expressive writing	There was a statistically significant reduction in GHQ-28 scores in both groups 4 weeks after the intervention, but not in psychiatric distress.	The effect of expressive writing remains equivocal when it comes to reducing psychological distress in vitiligo patients. The use of phototherapy may be associated with a decline in psychological distress.	No significant between-group differences in GHQ-28 total or sub-scores after 4 weeks (*p* = 0.7); significant within-group reductions observed in both groups (Group A: 47.9 ± 11.71, *p* = 0.000; Group B: 48.94 ± 10.69, *p* = 0.000).
**A novel, web-based, psychological intervention for people with psoriasis: the electronic Targeted Intervention for Psoriasis (eTIPs) study**	(Bundy et al., 2013) [28] Q1 (high)	To determine whether an electronic CBT intervention for psoriasis (eTIPs) would reduce distress, improve quality of life and clinical severity in patients with psoriasis.	Randomized study	Electronic, web-based Cognitive Behavioral Therapy intervention	Anxiety scores between groups were significantly reduced only for complete cases, depression scores did not change, as did psoriasis severity scores. Quality of life scores improved in both analyses.	This first online CBT intervention for people with skin conditions has shown improved anxiety and quality of life in patients with psoriasis.	Significant between-group reduction in anxiety scores (*p* = 0.004); no significant between-group differences in depression or psoriasis severity; significant between-group improvement in quality of life (*p* = 0.042).
**Mindfulness-Based Interventions for Psoriasis: a Randomized Controlled Trial**	(D’Alton et al., 2019b) [17] Q1 (high)	This study aims to compare the effectiveness of mindfulness-based cognitive therapy (MBCT), mindfulness-based self-compassion therapy (MBSCT) and self-help MBSCT (MBSCT-SH) compared to treatment as usual (TAU) in improving the long-term psychological and physical outcomes of individuals with psoriasis.	Randomized controlled trial.	Mindfulness-based cognitive therapy (MBCT), Mindfulness-based self-compassion therapy (MBSCT) and Self-help MBSCT (MBSCT-SH)	Although participants reported that MBIs were beneficial, no statistically significant differences were found in psychological well-being, severity of psoriasis symptoms or quality of life compared to TAU alone at post-treatment (follow-up 6 or 12 months).	There were no statistically significant differences between the MBIs in improving anxious or depressive symptoms, nor in increasing self-compassion.	No significant between-group differences in psychological well-being, psoriasis symptom burden, or quality of life were observed at post-treatment, 6-month, or 12-month follow-up;
**The skin expressing affection: a group intervention with patients suffering from psychodermatoses**	(Da Silva et al., 2011) [36] Q1 (high)	To analyze a group intervention as a tool to promote new channels for the expression of affections in patients with psychodermatoses being treated at a public dermatology outpatient clinic.	Exploratory and descriptive study of a qualitative nature	Intervention group	The results show that the group was able to become a space for listening to and accepting suffering, allowing participants to see themselves and others, as well as to rehearse movements and changes in the way they relate to themselves and others.	The conclusion is that group intervention can be an important tool in dealing with patients with psychodermatoses, since it highlights the emotional aspects of this disease, favoring a new perspective and a more integrated model of care.	Group intervention facilitated emotional processing, self-acceptance, and improved interpersonal awareness; qualitative improvements reported, but no quantitative between-group comparisons were provided
**An Open Trial on the Feasibility and Efficacy of a Mindfulness-Based Intervention with Psychoeducational Elements on Atopic Eczema and Chronic Itch**	(Harfensteller, 2022) [32] Q1 (high)	This article reports on a novel Mindfulness based Training for chronic Skin Conditions (MBTSC) with psychoeducational elements that was developed with the goal of improving self-regulation including stress management and emotion regulation in patients and to help in coping with disease symptoms such as itch and scratching.	Open trial	Mindfulness based Training for chronic Skin Conditions (MBTSC)	Quantitative data showed improvements in disease severity, itch perception and stress levels with small to medium effect sizes. Psychological distress increased at post-treatment—significantly in the case of depression. Qualitative data highlighted the mixed effects of MBTSC on symptoms. Treatment acceptability was high and 100% of the participants completed the intervention	These data indicate that MBTSC is feasible and that it might be a useful tool as adjunct therapy for AD. Further studies with larger samples and control groups are needed.	Moderate reductions in disease severity (PO-SCORAD: d = 0.65, *p* = 0.290) and itch perception (EIQ: d = 0.41, *p* = 0.407), with greater decrease in the affective-emotional dimension of itch (d = 0.51, *p* = 0.320, not significant); moderate improvements in subjective stress and mindfulness, not statistically significant; significant increase in depression (HADS-D: d = 1.43, *p* = 0.033) and no significant change in anxiety (HADS-A: d = 0.28, *p* = 0.530); high acceptability with 100% completion; subjective improvements reported in symptoms and disease coping; no between-group comparisons were performed.
**Exposure-based cognitive behavior therapy for atopic dermatitis: an open trial**	(Hedman-Lagerlöf et al., 2019) [30] Q1 (high)	The aim of the present study was to test the treatment’s acceptability and preliminary efficacy in adults with AD.	Pilot study	Exposure-based Cognitive–Behavioral Therapy for adult with Atopic Dermatitis	The results showed significant and large baseline to posttreatment improvements on self-reported measures of AD symptoms (*p* = 0.020) and general anxiety (*p* = 0.005), but there was no significant improvement in depression or quality of life. Treatment satisfaction was high, and most participants (67%) completed the treatment.	We conclude that exposure-based CBT for adult AD can be feasible, acceptable, and potentially efficacious.	Significant baseline-to-posttreatment improvements in AD symptoms (*p* = 0.020) and general anxiety (*p* = 0.005); no significant change in depression or quality of life; no between-group comparisons were performed.
**Compassion-focused self-help for psychological distress associated with skin conditions: a randomized feasibility trial**	(Hudson et al., 2020) [21] Q1 (high)	Test the feasibility of a self-help intervention based on Compassion-Focused Theory (CBT) and understand the effects of the treatment on a population of adults with skin diseases.	Randomized controlled study	Compassion-Focused Theory (CFT) self-help intervention	The CFT self-help intervention shows promise results in treating psychological distress associated with skin conditions.	Although the study indicates that the intervention may be promising in treating psychological distress associated with skin problems, further testing of the intervention is not feasible without significant methodological changes, including the way the treatment is administered.	Significant between-group improvements in stress, anxiety, depression, self-compassion, and dermatology-specific quality of life among completers (moderate effect sizes); effects remained significant in intention-to-treat (ITT) analyses but with reduced effect sizes (small); 51% completion rate; participants practiced a median of 9/14 days.
**Soothing Oneself and Resisting Self-Attacks: The Treatment of Two Intrapersonal Deficits in Depression Vulnerability**	(Kelly et al., 2009) [16] Q1 (high)	Test the impact of two self-help interventions designed to reduce depression in acne patients, by improving difficulties with self-soothing and resisting self-attacks.	Randomized controlled trial.	Self-help interventions: self-soothing and resist self-attacks	The results indicate that among acne sufferers, practicing a more calming style of self-talk can reduce shame and skin problems, just as practicing stronger posture and more resilient self-talk can reduce depression, shame and skin complaints.	In two weeks, the self-soothing intervention lowered shame and skin complaints. The attack-resisting intervention lowered depression, shame, and skin complaints, and was especially effective at lowering depression for self-critics.	Significant between-group improvements were observed in depression (attack-resisting vs. control, F (1,69) = 6.00, *p* = 0.02), shame (self-soothing: F (1,71) = 5.13, *p* = 0.03; attack-resisting: F (1,71) = 6.92, *p* = 0.01), and acne-related emotional distress (self-soothing: F (1,70) = 10.40, *p* < 0.01; attack-resisting: F (1,70) = 17.47, *p* < 0.001) compared to controls. Physical acne symptoms decreased significantly in the self-soothing condition (F (2,70)= 6.25, *p* = 0.01) with a trend for the attack-resisting condition (F (2,70) = 3.38, *p* = 0.07); no differences were found between interventions.
**Efficacy of Integrated Online Mindfulness and Self-compassion Training for Adults with Atopic Dermatitis: A Randomized Clinical Trial**	(Kishimoto et al., 2023) [20] Q1 (high)	To evaluate the efficacy of mindfulness and self-compassion training in improving the QOL for adults with AD.	Randomized clinical trial	Integrated Online Mindfulness and Self-compassion Training	Primary outcome: change in dermatology life quality score from baseline to week 13. Secondary outcomes: eczema severity, itch- and scratching-related visual analog scales, self-compassion and all of its subscales, mindfulness, psychological symptoms, and participants’ adherence to dermatologist-advised treatments.	These findings suggest that mindfulness and self-compassion training is an effective treatment option for adults with AD.	Significant between-group improvement in DLQI at 13 weeks (mean difference = −6.34, 95% CI −8.27 to −4.41, *p* < 0.001, Cohen’s d =–1.06); all secondary outcomes also showed greater improvements in the intervention group compared to the waiting list, although exact between-group statistics for these were not reported.
**A Factorial Randomized Controlled Trial of Implementation- Intention- Based Self-Affirmation Intervention: Findings on Depression, Anxiety and Well-being in Adults With Psoriasis**	(Łakuta, 2022) [24] Q1 (high)	Study whether strengthening the specificity element within the body-related self-affirmative implementation intention intervention compared to general self-affirmative implementation intention would provide greater improvements for adults with psoriasis.	Randomized Study	Implementation- Intention- Based Self-affirmation interventions	Exploratory analysis revealed two moderating effects of age and self-esteem, pointing to borderline conditions of the interventions.	These findings offer deeper insights into the negative effects also reported in previous work and highlight that self-affirmation interventions must be further investigated and optimized before they can be widely implemented in real-life contexts.	Significant within-group improvements over time were observed for depressive symptoms (F (2, 377) = 11.91, *p* < 0.001), anxiety (F (2, 380) = 30.82, *p* < 0.001), and well-being (F (2, 372) = 11.94, *p* < 0.001), but no significant between-group differences or time × group interactions were detected for depressive symptoms (F (4, 377) = 0.12, *p* = 0.978), anxiety (F (4, 380) = 1.41, *p* = 0.230), or well-being (F (4, 372) = 0.19, *p* = 0.942).
**A telephone-based motivational interviewing intervention has positive effects on psoriasis severity and self-management: a randomized controlled trial**	(Larsen et al., 2014b) [19] Q1 (high)	Evaluate the effects of a 3-month individual motivational interviewing intervention on psoriasis patients (with a total follow-up of 6 months) after climatherapy/heliotherapy (CHT).	Randomized controlled trial.	3-month individual motivational interviewing intervention	There were significant overall treatment effects in the study group in terms of the SAPASI score. The parameters of lifestyle change and knowledge about the disease were significantly better in the experimental group.	The results showed that the study group differed from the control group at 6 months after CHT in terms of disease severity, knowledge about psoriasis, self-efficacy and some lifestyles change parameters.	Significant between-group improvements were observed in the study group for SAPASI scores, three self-management domains of heiQ, and self-efficacy (*p* < 0.05). Lifestyle change parameters were also significantly better in the study group. Additionally, illness perception differed between groups at 3 months (*p* = 0.014), and psoriasis knowledge was significantly higher in the study group at 6 months (*p* = 0.017).
**Evaluation of the effectiveness of self-healing training on self-compassion, body image concern, and recovery process in patients with skin cancer**	(Latifi et al., 2020) [34] Q1 (high)	To investigate the effect of self-healing training on self-compassion, body image concern, and recovery process in patients with skin cancer.	Quasi-experimental; pre- and post-test with a control group	Self-healing training on self-compassion, body image concern, and recovery process	Self-healing training significantly increased self-compassion, including self-kindness, self-judgment, and sense of common humanity, and decreased the level of body image concern, isolation, and over-identification.	The self-healing is an appropriate intervention method to increase self-compassion and reduce body image concern and thus accelerate the process of skin cancer recovery.	Following self-healing training, significant increases were observed in self-compassion (self-kindness, self-judgment, and sense of common humanity; *p* < 0.01) and significant decreases in body image concern, isolation, and over-identification (*p* < 0.05)
**Efficacy of psychological intervention for patients with psoriasis vulgaris: a prospective study**	(Li et al., 2020) [35] Q3 (low)	To examine the effect of a psychological intervention on patients with psoriasis vulgaris.	Prospective study	Several	The study group showed significantly better SCL-90 and GQOLI scores than the non-psoriasis group. After treatment, they also had lower SDS and SAS scores, and higher satisfaction and compliance rates compared to the control group.	Psychological intervention may be beneficial for improving quality of life and the therapeutic efficacy of drugs in patients with psoriasis.	Significant between-group improvements were observed after treatment, with the study group showing better SCL-90 and GQOLI scores compared with the control group. Additionally, the study group had improved SDS (31.99 ± 4.54 vs. 44.08 ± 4.52) and SAS scores (28.36 ± 4.52 vs. 40.14 ± 6.33), as well as higher patient satisfaction and compliance rates.
**Feasibility and pilot study of a brief self-compassion intervention addressing body image distress in breast cancer survivors**	(Mifsud et al., 2021b) [22] Q1 (high)	Explore the feasibility and acceptability of the MyCB intervention, with and without an additional meditation component, in breast cancer survivors.	Randomized controlled study	Brief web-based self-compassion intervention MyCB, alone and with meditation	Adherence to MyCB writing and meditation was moderate, and acceptability was high for both MyCB and MyCB + M. Post-intervention state self-compassion and positive affect increased.	The results provide preliminary evidence for the efficacy and potential clinical use of the brief web-based self-compassion intervention MyCB, alone and with the addition of meditation, to increase self-compassion and psychological well-being in breast cancer survivors.	Post-intervention, MyCB (combined) showed increased self-compassionate attitude (F_1,23_ = 12.10, *p* = 0.002, d = 0.95) and positive affect (F_1,23_ = 4.34, *p* = 0.046, d = 0.83) compared to EW. At 1-month follow-up, body image distress decreased (F_1,23_ = 8.19, *p* = 0.009), trait self-compassion increased for MyCB + M vs. MyCB (t_23_ = 2.70, *p* = 0.013, d = 0.74), and anxiety decreased for MyCB + M vs. EW (t_23_ = −3.464, *p* = 0.002, d = 0.31) and MyCB (t_23_ = −3.893, *p* = 0.001, d = 0.22). No other between-group differences were significant at baseline or follow-up.
A randomized controlled feasibility trial of online compassion-focused self-help for psoriasis*	(Muftin et al., 2022) [23] Q2 (medium)	Test the feasibility and acceptability of two studies that theoretically developed self-help interventions designed to reduce feelings of shame and improve quality of life.	Randomized controlled study	Online compassion and mindfulness-focused self-help	Both interventions showed moderate but statistically significant reductions in shame and improvements in quality of life.	Self-help based on compassion and mindfulness is acceptable and can reduce feelings of shame and improve the quality of life for people living with psoriasis.	Both interventions were well accepted, with over 70% of completers reporting the materials were helpful and produced modest but significant reductions in shame (Cohen’s d = 0.20) and improvements in quality of life (Cohen’s d = 0.40); overall attrition was 29%.
**A Pilot Study of a Mindfulness-Based Stress Reduction Programme in Patients Suffering from Atopic Dermatitis**	(Offenbächer et al., 2021) [31] Q1 (high)	Assess the feasibility, acceptability, and effectiveness of a Mindfulness-Based Stress Reduction (MBSR) Program in patients with atopic dermatitis.	Pilot study	Mindfulness-Based Stress Reduction (MBSR) Programme	The IMF indicated significant improvement in the “presence” and “acceptance” subscales. There was also a tendency to less stress.	Considering the long history and the severity of the disease burden, the effects of this intervention appear promising as a complement to conventional treatments.	Pre-post analysis showed significant improvements in the FMI presence and ‘acceptance’ subscales. A tendency toward reduced stress was also observed. These findings were supported by participants’ qualitative statements.
**Impact of Psychological Intervention in Women with Alopecia Areata Universalis: a Pilot Study**	(Pascual-Sánchez et al., 2020) [29] Q3 (low)	To evaluate the usefulness of cognitive–behavioral therapy as a psychological intervention within a psychoeducational group setting for women with alopecia areata universalis (AAU)n, and to identify the key elements that can help improve the quality of care in this area.	Randomized pilot study.	Cognitive–behavioral therapy as a psychological intervention within a psychoeducational group	The intervention improved sleep and quality of life, though emotional difficulties like alexithymia increased. Anxiety and depression remained linked to well-being throughout.	The results suggest that psychological intervention can improve quality of life and sleep in women with AAU, key aspects of their well-being. While anxiety showed only slight improvement—possibly due to the short treatment duration—more complex issues like anxiety and depression may need longer interventions. The rise in alexithymia might indicate the early stages of emotional awareness. Future studies are needed to explore these findings further.	The psychological intervention in women with AAU resulted in significant improvements in quality of life (DLQI, pre-post comparison, *p* = 0.041) and sleep quality (OSQ, *p* < 0.01), alongside a paradoxical increase in alexithymia (TAS-20, *p* = 0.025). No significant changes were observed in depression, anxiety, or self-esteem.
**Psychological Therapies and Mind–Body Techniques in the Management of Dermatologic Diseases**	(Rafidi et al., 2022) [39] Q1 (high)	To determine the efficacy of various psychological therapies and mind–body techniques in the management of common dermatologic diseases in individuals of all ages.	Systematic Review	Various psychological therapies and mind–body techniques including cognitive–behavioral therapy (CBT), mindfulness-based interventions, and habit reversal therapy.	The studies focused mainly on psoriasis and atopic dermatitis, along with other skin conditions such as vitiligo, pruritus, and acne. The identified therapies showed positive effects on both physical symptoms and psychological well-being	There is a bidirectional relationship between skin diseases and psychological distress. Psychological therapies and mind–body techniques are effective adjunctive treatments in managing dermatologic diseases. The review highlights the potential benefits of integrating these interventions into standard care, especially for select patients	Based on the analysis of included studies and assessment of evidence quality, the most promising interventions were cognitive–behavioral therapy, mindfulness-based interventions, and habit reversal therapy.
**Feasibility assessment of an 8-week attention-based training programme in the management of chronic spontaneous urticaria**	(Ridge et al., 2021) [33] Q1 (high)	Developing a mindfulness-based training course for individuals with chronic spontaneous urticaria.	Prospective cutting study	Mindfulness-based training course (ABT programme)	A decrease in the severity of urticaria symptomatology as measured by the urticaria control test was observed after completion of the intervention.	Integration of an ABT program into routine clinical care for patients with chronic spontaneous urticaria is feasible and was considered acceptable and valuable by the individuals who participated.	Within-group improvements were observed in urticaria symptom severity, psychological well-being, and mindfulness practice adherence following the 8-week ABT programme; however, no between-group comparisons were reported, and participant retention was limited (33% dropout).
**The effectiveness of self-compassion and self-esteem writing tasks in reducing body image concerns**	(Seekis et al., 2017b) [1] Q1 (high)	Investigate whether single-session self-compassion and self-esteem writing tasks enrich the body image concerns evoked by a negative body image induction.	Randomized study.	Single-session self-compassion and self-esteem writing tasks	The self-compassion writing group showed higher post-treatment body appreciation and higher body satisfaction.	Writing-based interventions, especially those that enhance self-compassion, may help improve certain body image concerns.	Significant within-group improvement in body appreciation and body satisfaction was observed in the self-compassion writing group compared to control (post-treatment and 2-week follow-up), while no between-group differences were found for appearance anxiety.
**Mindful self-compassion for psychological distress associated with skin conditions: An online intervention study**	(Sengupta et al., 2025) [25] Q2 (medium)	To evaluate the impact of a Mindful Self-Compassion (MSC) intervention on depression, anxiety, stress, self-esteem, well-being, and dermatology-specific quality of life in adults with chronic skin conditions.	Randomized controlled study	Mindful Self-Compassion (MSC) a structured mindfulness-based self-compassion training program incorporating elements of personal development and psychotherapy, delivered online	The study reported statistically significant improvements in the intervention group, with reduced levels of depression, anxiety, and stress, as well as increased self-esteem, well-being, and dermatology-specific quality of life when compared to the waitlist control group	MSC was shown to be an effective approach for managing psychological distress associated with chronic skin conditions.	Significant between-group improvements were observed in depression (*p* < 0.001), anxiety (*p* < 0.001), stress (*p* < 0.001), dermatology-related quality of life (*p* < 0.001), self-esteem (*p* < 0.001), and well-being (*p* < 0.001) in the mindful self-compassion intervention group compared to waitlist control.
**Enhancing self-compassion in individuals with visible skin conditions: randomized pilot of the ‘My Changed Body’ self-compassion writing intervention**	(Sherman et al., 2019) [26] Q1 (high)	Investigate the feasibility of applying the My Changed Body intervention to address visible body image issues related to the skin.	Randomized pilot study.	Self-compassion writing intervention “My Changed Body”	Self-compassion and negative affect showed improvements in the experimental group compared to the control group. There was no between groups difference at follow-up in positive affect.	The My Changed Body writing intervention may provide benefit to individuals with visible skin condition.	Significant between-group improvements were observed in self-compassion (*p* = 0.006) and reductions in negative affect (*p* = 0.028) in the self-compassion writing intervention group compared to control, while no significant between-group differences were found for positive affect.
**Clinic-based Group Multi-professional Education Causes Significant Decline in Psoriasis Severity: A Randomized Open Label Pilot Study**	(Singh et al., 2017) [18] Q3 (low)	Test the benefits of a multidisciplinary group intervention using psychoeducation.	Randomized controlled trial.	Multidisciplinary group intervention using psychoeducation	After the intervention, there was a statistically significant improvement in the mean scores on the PASI, DLQI and WHO-5 in the experimental group, unlike what was observed in the control group. There was a statistically significant improvement in PHQ 9 scores in both groups. The scores on the PHQ 15 and the GAD 7 showed no statistically significant differences.	Psychoeducational intervention group demonstrated improvements in clinical and psychological outcomes in patients with psoriasis.	Significant improvements in PASI, DLQI, and WHO-5 were observed in the intervention group at 6 months post-intervention (*p* < 0.01), while PHQ-9 scores improved in both intervention and control groups (*p* < 0.01). No significant changes were observed for PHQ-15 or GAD-7, and between-group differences were not reported
**Integrative Treatment Approaches with Mind–Body Therapies in the Management of Atopic Dermatitis**	(Yosipovitch et al., 2024) [8] Q1 (high)	To highlight the role of psychodermatology in atopic dermatitis by exploring mind–body therapies that address the psychological factors influencing the severity and management of the disease.	Focused literature review	Cognitive–behavioral therapy, mindfulness, meditation, hypnotherapy, habit reversal, relaxation techniques, EMDR, music therapy, massage, education, and other mind–body therapies.	Mind–body therapies may reduce pruritus, psychological stress, anxiety, depressive symptoms, pain, and improve sleep and adherence to treatment in patients with atopic dermatitis.	Mind–body therapies show potential benefits as adjunct treatments for atopic dermatitis, but more rigorously designed trials are needed to confirm long-term efficacy	Integrative mind–body therapies (MBT), when combined with conventional pharmacologic treatment, show potential benefits for patients with atopic dermatitis, including reductions in pruritus, pain, psychological stress, anxiety, depressive symptoms, and improvements in sleep quality and treatment adherence.
**Patients’ Experiences of an Acceptance and Commitment Therapy-Based Approach for Psychosocial Difficulties Relating to an Appearance-Affecting Condition**	(Zucchelli et al., 2021) [37] Q1 (high)	Investigate the lived experiences of patients with various dermatological conditions and appearance-related concerns who took part in Acceptance and Commitment Therapy sessions.	Qualitative study.	Acceptance and Commitment Therapy (ACT) -Based Approach	ACT-based individual therapy seemed to help speed up the process of accepting a changed appearance, which the participants highlighted as an important factor.	Participants emphasized the importance of therapists expressing compassion and helping patients cultivate self-compassion in their daily lives.	ACT-based individual therapy improved acceptance of appearance and fostered body confidence and self-compassion, as reported by participants.

^1^ According to Scimago Journal & Country Rank.

## Data Availability

The original contributions presented in this study are included in the article/Supplementary Materials. Further inquiries can be directed to the corresponding authors.

## Supplementary Materials

The following supporting information can be downloaded at: https://www.mdpi.com/article/10.3390/healthcare13222947/s1, Table S1: PRISMA 2020 Checklist.

## Abbreviation

CBT	Cognitive–Behavioral Therapy

## References

[1] Seekis V., Bradley G.L., Duffy A. (2017). The effectiveness of self-compassion and self-esteem writing tasks in reducing body image concerns. Body Image.

[2] Ücker Calvetti P., Rivas R., Coser J., Barbosa A., Ramos D. (2017). Biopsychosocial aspects and quality of life of people with chronic dermatoses. Psicol. Saúde Doença.

[3] Clarke E.N., Thompson A.R., Norman P. (2020). Depression in people with skin conditions: The effects of disgust and self-compassion. Br. J. Health Psychol..

[4] Rumsey N., Harcourt D. (2004). Body image and disfigurement: Issues and interventions. Body Image.

[5] Thompson A., Kent G. (2001). Adjusting to disfigurement: Processes involved in dealing with being visibly different. Clin. Psychol. Rev..

[6] Misery L., Schut C., Balieva F., Bobko S., Reich A., Sampogna F., Altunay I., Dalgard F., Gieler U., Kupfer J. (2023). White paper on psychodermatology in Europe: A position paper from the EADV Psychodermatology Task Force and the European Society for Dermatology and Psychiatry (ESDaP). J. Eur. Acad. Dermatol. Venereol..

[7] Chen Y., Lyga J. (2014). Brain-skin connection: Stress, inflammation and skin aging. Inflamm. Allergy-Drug Targets.

[8] Yosipovitch G., Bernhard J.D. (2013). Chronic pruritus. N. Engl. J. Med..

[9] Ahmed A., Steed L., Burden-Teh E., Shah R., Sanyal S., Tour S., Dowey S., Whitton M., Batchelor J.M., Bewley A.P. (2018). Identifying key components for a psychological intervention for people with vitiligo—A quantitative and qualitative study in the United Kingdom using web-based questionnaires of people with vitiligo and healthcare professionals. J. Eur. Acad. Dermatol. Venereol..

[10] Galhardo A., Mendes R., Massano-Cardoso I., Cunha M. (2022). Processos relacionados com a regulação emocional e vergonha e sua associação com os sintomas de depressão, ansiedade e stresse em pessoas com psoríase. Psychologica.

[11] Berry M.P., Lutz J., Schuman-Olivier Z., Germer C., Pollak S., Edwards R.R., Gardiner P., Desbordes G., Napadow V. (2020). Brief Self-Compassion Training Alters Neural Responses to Evoked Pain for Chronic Low Back Pain: A Pilot Study. Pain Med..

[12] Melissant H.C., Jansen F., Eerenstein S.E.J., Cuijpers P., Lissenberg-Witte B.I., Sherman K.A., Laan E.T.M., Leemans C.R., Verdonck-de Leeuw I.M. (2021). A structured expressive writing activity targeting body image-related distress among head and neck cancer survivors: Who do we reach and what are the effects?. Support. Care Cancer.

[13] Sherman K.A., Przezdziecki A., Alcorso J., Kilby C.J., Elder E., Boyages J., Koelmeyer L., Mackie H. (2018). Reducing Body Image–Related Distress in Women with Breast Cancer Using a Structured Online Writing Exercise: Results from the My Changed Body Randomized Controlled Trial. J. Clin. Oncol..

[14] Joanna Briggs Institute (2024). JBI Critical Appraisal Tools: Checklists for Assessing the Trustworthiness, Relevance and Results of Published Papers. https://jbi.global/critical-appraisal-tools.

[15] Adkins K.V., Overton P.G., Thompson A.R. (2022). A brief online writing intervention improves positive body image in adults living with dermatological conditions. Front. Med..

[16] Kelly A.C., Zuroff D.C., Shapira L.B. (2009). Soothing Oneself and Resisting Self-Attacks: The Treatment of Two Intrapersonal Deficits in Depression Vulnerability. Cogn. Ther. Res..

[17] D’Alton P., Kinsella L., Walsh O., Sweeney C., Timoney I., Lynch M., O’Connor M., Kirby B. (2019). Mindfulness-Based Interventions for Psoriasis: A Randomized Controlled Trial. Mindfulness.

[18] Singh S.M., Narang T., Vinay K., Sharma A., Satapathy A., Handa S., Dogra S. (2017). Clinic-based group multi-professional education causes significant decline in psoriasis severity: A randomized open label pilot study. Ind. Dermatol. Online J..

[19] Larsen M.H., Krogstad A.L., Aas E., Moum T., Wahl A.K. (2014). A telephone-based motivational interviewing intervention has positive effects on psoriasis severity and self-management: A randomized controlled trial. Br. J. Dermatol..

[20] Kishimoto S., Watanabe N., Yamamoto Y., Imai T., Aida R., Germer C., Tamagawa-Mineoka R., Shimizu R., Hickman S., Nakayama Y. (2023). Efficacy of integrated online mindfulness and self-compassion training for adults with atopic dermatitis: A randomized clinical trial. JAMA Dermatol..

[21] Hudson M.P., Thompson A.R., Emerson L.-M. (2020). Compassion-focused self-help for psychological distress associated with skin conditions: A randomized feasibility trial. Psychol. Health.

[22] Mifsud A., Pehlivan M.J., Fam P., O’Grady M., van Steensel A., Elder E., Gilchrist J., Sherman K.A. (2021). Feasibility and pilot study of a brief self-compassion intervention addressing body image distress in breast cancer survivors. Health Psychol. Behav. Med..

[23] Muftin Z., Gilbert P., Thompson A.R. (2022). A randomized controlled feasibility trial of online compassion—focused self-help for psoriasis. Br. J. Dermatol..

[24] Łakuta P. (2022). A Factorial Randomized Controlled Trial of Implementation-Intention-Based Self-Affirmation Interventions: Findings on Depression, Anxiety, and Well-being in Adults with Psoriasis. Front. Psychiatry.

[25] Sengupta A., Wagani R. (2024). Mindful self-compassion for psychological distress associated with skin conditions: An online intervention study. Ind. J. Dermatol. Venereol. Leprol..

[26] Sherman K.A., Roper T., Kilby C.J. (2019). Enhancing self-compassion in individuals with visible skin conditions: Randomised pilot of the ‘My Changed Body’ self-compassion writing intervention. Health Psychol. Behav. Med..

[27] Borimnejad L., Firooz A., Mortazavi H., Aghazadeh N., Halaji Z. (2015). The Effect of Expressive Writing on Psychological Distress in Patients with Vitiligo: A Randomized Clinical Trial. J. Client-Centered Nurs. Care.

[28] Bundy C., Pinder B., Bucci S., Reeves D., Griffiths C.E.M., Tarrier N. (2013). A novel, web-based, psychological intervention for people with psoriasis: The electronic Targeted Intervention for Psoriasis (eTIPs) study. Br. J. Dermatol..

[29] Pascual-Sánchez A., Fernández-Martín P., Saceda-Corralo D., Vañó-Galván S. (2020). Impact of psychological intervention in women with alopecia areata universalis: A pilot study. Actas Dermo-Sifiliogr..

[30] Hedman-Lagerlöf E., Bergman A., Lindefors N., Bradley M. (2019). Exposure-based cognitive behavior therapy for atopic dermatitis: An open trial. Cogn. Behav. Ther..

[31] Offenbächer M., Seitlinger M., Münch D., Schnopp C., Darsow U., Harfensteller J., Schmid-Grendelmeier P., Ring J., Kohls N. (2021). A Pilot Study of a Mindfulness-Based Stress Reduction Programme in Patients Suffering from Atopic Dermatitis. Psych.

[32] Harfensteller J. (2022). An Open Trial on the Feasibility and Efficacy of a Mindfulness-Based Intervention with Psychoeducational Elements on Atopic Eczema and Chronic Itch. Psych.

[33] Ridge K., Conlon N., Hennessy M., Dunne P.J. (2021). Feasibility assessment of an 8-week attention-based training programme in the management of chronic spontaneous urticaria. Pilot Feasibility Stud..

[34] Latifi Z., Soltani M., Mousavi S. (2020). Evaluation of the effectiveness of self-healing training on self-compassion, body image concern, and recovery process in patients with skin cancer. Complement. Ther. Clin. Pract..

[35] Li X., Liu L., Zhang Y., Li L. (2020). Efficacy of psychological intervention for patients with psoriasis vulgaris: A prospective study. J. Int. Med. Res..

[36] Da Silva A., Castoldi L., Kijner L. (2011). A pele expressando o afeto: Uma intervenção grupal com pacientes portadores de psicodermatoses. Contextos Clínicos.

[37] Zucchelli F.A., Donnelly O., Sharratt N.D., Hooper N., Williamson H.M. (2021). Patients’ Experiences of an Acceptance and Commitment Therapy-Based Approach for Psychosocial Difficulties Relating to an Appearance-Affecting Condition. Eur. J. Couns. Psychol..

[38] Bartholomew E., Chung M., Yeroushalmi S., Hakimi M., Bhutani T., Liao W. (2022). Mindfulness and meditation for psoriasis: A systematic review. Dermatol. Ther..

[39] Rafidi B., Kondapi K., Beestrum M., Basra S., Lio P. (2022). Psychological therapies and mind–body techniques in the management of dermatologic diseases: A systematic review. Am. J. Clin. Dermatol..

[40] Nguyen T.T., Jensen C.G., Khoury L., Deleuran B., Blom E.S., Breinholt T., Christensen R., Skov L. (2021). Effectiveness of mind–body intervention for inflammatory conditions: Results from a 26-week randomized, non-blinded, parallel-group trial. J. Clin. Med..

[41] Hewitt R.M., Ploszajski M., Purcell C., Pattinson R., Jones B., Wren G.H., Hughes O., Ridd M.J., Thompson A.R., Bundy C. (2022). A mixed methods systematic review of digital interventions to support the psychological health and well-being of people living with dermatological conditions. Front. Med..

[42] Hughes O., Shelton K.H., Penny H., Thompson A.R. (2023). Parent and child experience of skin conditions: Relevance for the provision of mindfulness-based interventions. Br. J. Dermatol..

[43] Hopwood P., Fletcher I., Lee A., Al Ghazal S. (2001). A body image scale for use with cancer patients. Eur. J. Cancer.

[44] Fried R.G., Gupta M.A., Gupta A.K. (2005). Depression and skin disease. Dermatol. Clin..

[45] Almeida V., Leite Â., Constante D., Correia R., Almeida I.F., Teixeira M., Vidal D.G., e Sousa H.F.P., Dinis M.A.P., Teixeira A. (2020). The Mediator Role of Body Image-Related Cognitive Fusion in the Relationship between Disease Severity Perception, Acceptance and Psoriasis Disability. Behav. Sci..

[46] Ziemer K.S., Lamphere B.R., Raque-Bogdan T.L., Schmidt C.K. (2019). A randomized controlled study of writing interventions on college women’s positive body image. Mindfulness.

[47] Türk K.E., Yılmaz M. (2018). The effect on quality of life and body image of mastectomy among breast cancer survivors. Eur. J. Breast Health.

[48] Birdi G., Cooke R., Knibb R.C. (2020). Impact of atopic dermatitis on quality of life in adults: A systematic review and meta-analysis. Int. J. Dermatol..

[49] Black D.S., Slavich G.M. (2016). Mindfulness meditation and the immune system: A systematic review of randomized controlled trials. Ann. N. Y. Acad. Sci..

[50] Dunn T.J., Dimolareva M. (2022). The effect of mindfulness-based interventions on immunity-related biomarkers: A comprehensive meta-analysis of randomised controlled trials. Clin. Psychol. Rev..

